# Predicting Tacrolimus Concentrations in the Skin of Adult Kidney Transplant Recipients: A Feasibility Study

**DOI:** 10.3389/ti.2024.12019

**Published:** 2024-01-23

**Authors:** Felicity Sartain, Andrea K. Viecelli, Margaret Veitch, Michael E. Franklin, Brian W. Dymock, James W. Wells, Scott B. Campbell

**Affiliations:** ^1^ Department of Medicine, Cairns Hospital, Cairns, QLD, Australia; ^2^ Department of Kidney and Transplant Services, Princess Alexandra Hospital, Brisbane, QLD, Australia; ^3^ Frazer Institute, Faculty of Medicine, The University of Queensland, Brisbane, QLD, Australia; ^4^ Department of Clinical Pharmacology, Princess Alexandra Hospital, Brisbane, QLD, Australia; ^5^ Queensland Emory Drug Discovery Initiative, UniQuest, The University of Queensland, Brisbane, QLD, Australia; ^6^ Dermatology Research Centre, Frazer Institute, Faculty of Medicine, The University of Queensland, Brisbane, QLD, Australia; ^7^ Faculty of Medicine, The University of Queensland, Brisbane, QLD, Australia

**Keywords:** kidney transplantation, organ transplant, skin cancer, tacrolimus, drug concentration, skin, calcineurin inhibitors

## Abstract

Solid organ transplant recipients are at an increased risk of developing skin cancers due to chronic immunosuppression, particularly with calcineurin inhibitors. Tacrolimus is the most prescribed calcineurin inhibitor in this patient cohort, and understanding tacrolimus concentrations in the skin will facilitate the development of anti-cancer preventive and therapeutic strategies. Here, we show that in mice, tacrolimus blood levels peaked rapidly ∼1 h post last oral dose while skin levels rose more slowly and remained high for at least 6 h. Subsequently, tacrolimus skin and blood concentrations were assessed in 15 kidney transplant recipients. The mean age was 61 years, the average time post-transplant was 7 years (range 0–21 years) and 87% were male. The average skin sampling time post tacrolimus dosing was 6 h 32 min. Skin tacrolimus concentrations ranged from 7.1 ng/g to 71.2 ng/g and correlated with blood concentrations (r = 0.6). Mouse and human mean skin concentrations were in a similar range. Our data suggests that tacrolimus measurements in the blood may be used to approximate tacrolimus concentrations in the skin of kidney transplant recipients, and further exploited for the delivery of anti-cancer therapies designed to antagonize the immunosuppressive effects of tacrolimus in the skin.

## Graphical Abstract

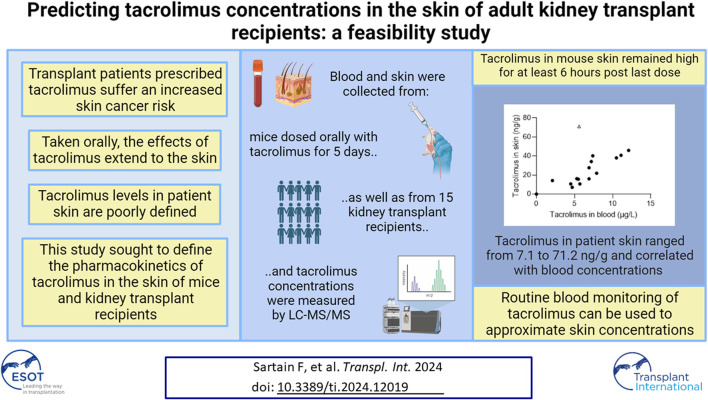

## Introduction

Solid organ transplant recipients are at an increased risk of developing malignancies as a consequence of their immunosuppression, with calcineurin inhibitors thought to be particularly responsible [1–3]. Skin cancer is the most common cancer type in kidney transplant recipients [4, 5]. The pathogenesis of skin cancer in transplant recipients involves predisposing risk factors and this is amplified by the carcinogenic effect of immunosuppressive medications. For instance, calcineurin inhibitors impair the capacity of the immune system to repair or destroy ultraviolet damaged cells [6]. Current pharmacologic therapies to prevent occurrence of skin cancers include retinoid therapy, nicotinamide and the modulation of immunosuppression by converting from calcineurin inhibitors to mammalian target of rapamycin (mTOR) pathway inhibitors [5, 7–9].

Tacrolimus, a calcineurin inhibitor, is commonly used to prevent rejection in solid organ transplants and acts by preventing the transcription of key pro-inflammatory cytokines within T cells which are necessary to drive an effective immune response [10, 11]. Tacrolimus also inhibits the presentation of exogenous antigens through the inhibition of antigen processing pathways and significantly inhibits helper T cell differentiation and cytokine secretion by CD4 memory T cells [3]. Nucleotide excision repair is inhibited by calcineurin inhibitors whereas calcineurin overexpression enhances cellular nucleotide excision repair [12]. Given that tacrolimus inhibits the capacity for ultraviolet-induced DNA repair, it is hypothesized that the inhibition of tacrolimus effects on cells in the skin may improve the ability of Sun damaged cutaneous cells to repair and thus may reduce the development of skin cancer. Importantly, this could happen without impeding the important systemic effects that tacrolimus has on the prevention of transplant rejection.

Recently, we have described the development of a novel and competitive tacrolimus inhibitor, Q-2361 [13]. Q-2361 is a reversible antagonist of the tacrolimus-FKBP12 binding interaction. The tacrolimus-FKBP12 complex binds to calcineurin forming a ternary complex thereby inhibiting calcineurin. A 400-1000-fold concentration of Q-2361 over tacrolimus facilitates human T cell function in the presence of tacrolimus. Transplant patients are known to have normal numbers of T cells in their skin despite many years of immunosuppression [14], and the local application of Q-2361 to squamous skin cancers growing in tacrolimus-suppressed mice has been shown to lead to T cell-mediated tumor rejection [13]. Thus, to progress the topical application of this compound towards clinical studies in patients, it is important to understand the concentration range of tacrolimus in patient skin compared to mouse skin and whether tacrolimus patient skin concentrations can be approximated from routine blood measurements of tacrolimus. Therefore, we aimed to test the hypotheses that it is possible to measure tacrolimus concentrations in the skin of adult kidney transplant recipients and that skin measurements correlate closely with blood measurements.

## Patients and Methods

### Mice

All animal procedures were approved by the University of Queensland Animal Ethics Committee; Approval Number UQDI/512/17. C57BL/6J mice were purchased from the Animal Resources Facility (Perth, Australia). All mice used were 12-week females and were housed under specific pathogen-free conditions at the Translational Research Institute Biological Research Facility (Brisbane, Australia).

### Oral Dosing With Tacrolimus in Mice

Mice were dosed orally with tacrolimus (MedChemExpress, Monmouth Junction, NJ, USA; 1 mg/kg) twice per day with a 7-hour interval for 4 days via oral gavage. On Day 5 the mice were orally dosed, and then cardiac bleeds and skin harvests were performed at the indicated timepoints. 110 μL of blood was transferred to a cryovial containing 10 µL 0.5M EDTA, shaken, snap-frozen on dry ice, and stored at −20°C. Approximately 2 cm^2^ of back skin was harvested, weighed, snap-frozen on dry ice, and stored at −20°C. The quantification of tacrolimus in blood was performed as previously described [15].

### Patient Study Setting and Design

Ethics approval was gained from the Metro South Human Research Ethics Committee (Approval number: HREC/2019/QMS/50547). Written consent from each adult kidney transplant recipient for involvement in this project was obtained prior to the removal of their presumed skin cancer. Eligible patients were adult kidney transplant recipients (≥18 years of age) who were on once or twice daily tacrolimus dosage and were planned to undergo a skin excision. Patients were excluded if they had a bleeding disorder or if they were on any anticoagulation other than aspirin. Included patients needed to have a planned surgical procedure which was likely to result in excess skin being available for sampling.

### Skin Sampling

Suspicious skin lesions were excised by the surgical team. Two 2–3 mm punch biopsies were immediately taken from the ends of the excised skin sample by the study investigators and placed on ice for transport to the laboratory (10 min). Once in the laboratory the skin biopsies were weighed, snap-frozen on dry ice, and stored at −20°C. The patients also had a blood tacrolimus level collected the same day of the excision, which was sent directly to the hospital clinical pharmacology department for assessment.

### Skin Tacrolimus Quantification

The quantification of tacrolimus in skin was performed as described [15]. Briefly, samples were placed in a tissue grinding tube (Precellys^®^ Lysing Kit; MK28-R; Bertin Technologies, Montigny-le-Bretonneux, France) containing 1 mL of Titrisol buffer (Merck KGaA, Darmstadt, Germany) and 60 µL of internal standard (ascomycin; Fujisawa Pharmaceutical Company, Osaka, Japan). Samples were homogenized using a Precellys^®^ 24 Homogenizer (Bertin Technologies) at 6,500 rpm, 30 s for 5 cycles, with the tubes placed on ice for at least 1 min between each cycle to prevent drug degradation. 5 mL tert-butyl methyl ether was added to each sample, and the liquid-liquid extraction process was performed manually by inverting the tubes for at least 5 min. Tubes were then centrifuged at room temperature at 1,800 g for 3 min to separate all the layers, and the tert-butyl methyl ether fraction containing partitioned tacrolimus was collected and evaporated using a Sample Concentrator (Techne Dri-Block, DB-3D, Cambridge, England) at 35°C and the residue subsequently reconstituted with 200 µL 50% methanol by vortexing thoroughly. Reconstituted samples were transferred into UPLC max recovery sample vials (Waters Corporation, Milford, MA) and analyzed using LC-MS/MS (Alliance HT LC system interfaced to a Quattro Micro tandem mass spectrometer; Waters Corporation) in the hospital clinical pharmacology department. Excess skin retrieved post abdominoplasty from a patient not on tacrolimus was used as a control.

### Statistical Analysis

Statistical and Pearson’s correlation coefficient analysis were carried out using GraphPad Prism version 9.4.1 (GraphPad Software, San Diego, CA, USA). There was no power calculation attached to the number of participants. Sample collection was concluded following visual evidence of a correlation between blood and skin tacrolimus levels.

## Results

### Pharmacokinetics of Tacrolimus in Mouse Skin

**FIGURE 1:**
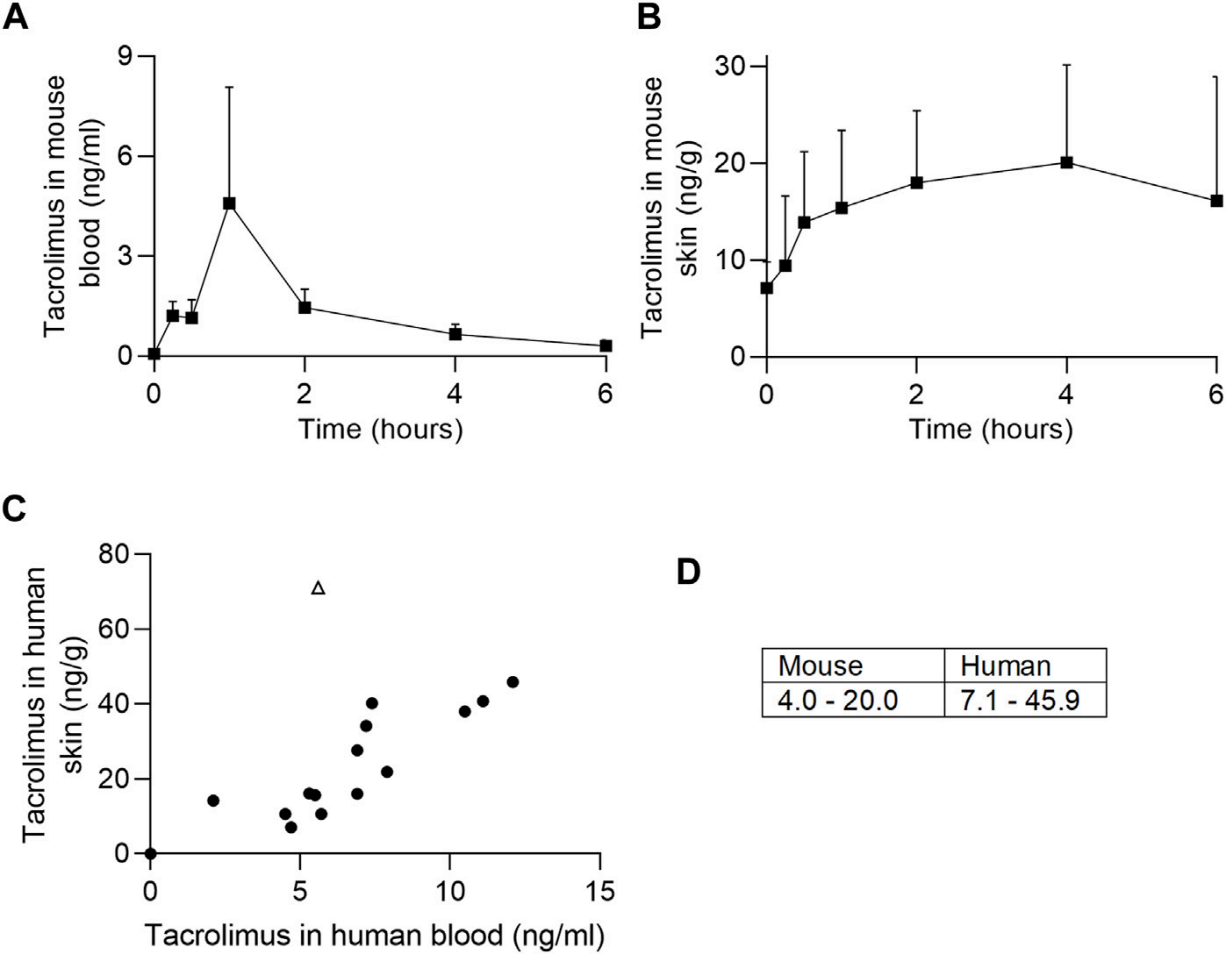
Tacrolimus assessments in blood and skin. **(A, B)** Tacrolimus levels in mouse blood and skin (respectively) following 4 days of twice-per-day oral dosing. *n* = 4 mice/timepoint, error bars represent standard error of the mean. **(C)** Comparison of tacrolimus levels in patient blood and skin. The outlier (pictured as an open triangle) was from a skin sample taken above an upper lip (tacrolimus skin concentration 71.2 ng/g). Correlations are shown with (r = 0.6, *p* < 0.05) and without (r = 0.88, *p* < 0.0001) outlier. **(D)** Mouse and human mean tacrolimus concentration data ranges in the skin (ng/g), excluding one human outlier with tacrolimus skin concentration of 71.2 ng/g.

To understand how tacrolimus concentrations in the blood and skin change at defined time points post oral dosing, we administered tacrolimus orally to mice twice daily over 5 days. At defined time points post last dose blood and skin were harvested and tacrolimus concentrations assessed by liquid chromatography with tandem mass spectrometry. As shown in Figure 1, tacrolimus blood levels peaked rapidly approximately 1 h post last oral dose (Figure 1A) while skin levels rose more slowly and remained high for at least 6 h (Figure 1B). The data suggests that skin tacrolimus levels do not rise and fall as quickly as they do in blood, and remained high for at least 6 h post last dose.

### Pharmacokinetics of Tacrolimus in Kidney Transplant Recipient Skin

Thirty-one patients were approached and consented. Of the thirty-one patients consented only fifteen patients proceeded to have skin lesions excised and thus were included in the study. Of the fifteen patients consented two (13%) were female and thirteen (87%) were male. The mean age was 61 years (range 48–72 years) and the average time since first transplant was 7 years (0–21 years). The majority of the patients (87%) were on the immunosuppressive regimen of tacrolimus, mycophenolate and prednisone. The average time post oral tacrolimus dosing was 6 h 32 min. Four of the patients had been treated for previous rejection. Two patients had had a second kidney transplant. Baseline characteristics of the patients who were included in the study are provided in Table 1.

**TABLE 1:** Baseline characteristics of study participants.

#	Sex	Age	Kidney disease	Year	Medication	Type and duration of dialysis	Weight (kg)
1	M	55	Reflux Nephropathy	2007	Tac, Aza, Pred	HD – 4 months	65.2
2	M	51	IgAN	2015	Tac, Lef, Pred	Pre-emptive	85
3	M	54	Renovascular Disease	2008	Tac, Myco, Pred	PD – uncertain duration	59
4	F	58	GN	2008, 2014	Tac, Myco, Pred	PD – 2 years btw transplants	96
5	M	59	Glomerulonephritis	2010	Tac, Myco, Pred	HD – uncertain duration	101.75
6	M	48	Lupus Nephritis	1998, 2005	Tac, Myco, Pred	HD – 13 years	83
7	M	65	PCKD	2019	Tac, Myco, Pred	HD – 1.5 years	95
8	M	56	IgAN/HSP	2010	Tac, Myco, Pred	Unknown	91
9	M	69	ADPKD	2005	Tac, Myco, Pred	HD – 1 year	71
10	M	60	Uncertain	2014	Tac, Myco, Pred	PD – 2.5 years	105
11	M	68	Nephritis	2012	Tac, Myco, Pred	PD – 2.5 years	102
12	F	69	ADPKD	2019	Tac, Myco, Pred	PD – 2.5 years	56.35
13	M	65	Post lung transplant, ATN	2017	Tac, Myco, Pred	HD – 5 years	95
14	M	72	Glomerulonephritis	2014	Tac, Myco, Pred	HD – 1.5 years	119.2
15	M	60	Caroli’s Disease	2013	Tac, Myco, Pred	HD – <1 year	94

#, patient number; M, Male; F, Female; IgAN, IgA Nephropathy; PCKD, Polycystic Kidney Disease; ADPKD, Autosomal polycystic kidney disease; GN, Glomerulonephritis unspecified; ATN, Acute Tubular Necrosis; Tac, Tacrolimus; Aza, Azathioprine; Myco, Mycophenolate; Lef, Leflunomide; Pred, Prednisone; HD, Haemodialysis; PD, Peritoneal Dialysis; HSP, Henoch Schoenlein Purpura; Year, year of transplant.

Twelve patients had an identifiable cutaneous malignancy on histopathology. Of the 23 samples obtained, 5 were classified as basal cell carcinomas, 4 samples were squamous cell carcinomas, 9 were intraepidermal carcinomas and 3 were solar keratoses. Two samples did not contain any pre-malignant or malignant tissue. These samples were taken from a variety of different anatomical locations as listed in Table 2.

**TABLE 2:** Tacrolimus measurements in blood and skin.

#	Total daily dose of Tac	Time since last dose (Hrs:Mins)	Time between samples (mins)	Tac in blood (ng/mL)	Skin Biopsy site, Tac conc (ng/g)	Mean Tac skin conc (ng/g)
1	2 mg BD (4 mg daily)	6:53	67^a^	11.1	Right lateral thigh, 40.8	40.8
2	6 mg BD (12 mg daily)	6:50	10^b^	5.6	Left upper lip, 71.2	71.2
3	1 mg BD (2 mg daily)	7:30	15^b^	5.5	Left forearm, 15.7	15.7
4	0.5 mg BD (1 mg daily)	9:35	7^b^	2.1	Left hand, 14.2	14.2
5	4 mg BD (8 mg daily)	5:35	10^a^	6.9	Neck, 27.0	27.7 (±0.9)
					Left shoulder, 28.4	
6	4.5 mg BD (9 mg daily)	4:40	5^a^	4.5	Right shoulder, 8.6	10.7 (±2.9)
					Right calf, 12.8	
7	1 mg mane/2 mg nocte (3 mg daily)	7:05	15^a^	4.7	Back, 7.1	7.1
8	1 mg mane/0.5 mg nocte (1.5 mg daily)	7:20	20^a^	10.5	Left ear, 38.0	38.0
9	1 mg BD (2 mg daily)	Unknown	5^a^	5.3	Right calf, 16.2	16.2
10	1 mg BD (2 mg daily)	8:10	15^b^	7.4	Left side of nose, 40.2	40.2
11	0.5 mg mane, 1 mg nocte (1.5 mg daily)	5:30	20^a^	7.9	Right forearm #1, 28.3	21.9 (±4.9)
					Right forearm #2, 17.6	
					Left lower calf, 23.4	
					Left shoulder, 18.5	
12	5 mg BD (10 mg daily)	4:45	30^a^	12.1	Left dorsum of hand, 38.9	45.9 (±9.8)
					Forehead, 52.9	
13	2 mg mane, 1.5 mg nocte (3.5 mg daily)	7:30	35^b^	6.9	Right knee, 16.1	16.1
14	0.5 mg mane, 1 mg nocte (1.5 mg daily)	6:45	8^b^	5.7	Right knee, 10.7	10.7
15	1.5 mg mane, 1 mg nocte (2.5 mg daily)	10:00	29^a^	7.2	Left cheek, 38.7	34.2 (±5.7)
					Left intra orbital, 36.3	
					Right forearm, 27.7	

#, patient number.

^a^ Blood sample taken before skin sample.

^b^ Skin sample taken before blood sample; Tac, Tacrolimus. Numbers in brackets in right hand column represent standard deviation.

The timing of skin excision was documented for all the patients involved in the study. The mean time between the skin sample excision and tacrolimus blood collection was 18.5 min (range 5–67 min). Five patients had multiple excisions taken and the tacrolimus cutaneous concentration was measured independently in all the specimens. Excess cutaneous tissue post abdominoplasty from a patient not taking tacrolimus was sent to the laboratory as a control. The tacrolimus concentration was 0 ng/g in this sample. Tacrolimus was detectable in the skin in all patients on oral tacrolimus.

The skin concentration of tacrolimus was calculated in twenty-three samples from fifteen patients (Table 2). Skin tacrolimus concentrations ranged from 7.1 ng/g to 71.2 ng/g. There was one clear outlier in the data: patient number two had a tacrolimus blood concentration of 5.6 μg/L and the concentration obtained from the skin sample was 71.2 ng/g. In patients in whom multiple skin samples were taken for tacrolimus skin concentration measurement, the mean tacrolimus concentration was used for the correlation calculations. The blood concentration of tacrolimus correlated with the concentration of tacrolimus detected in the skin samples (Figure 1C) with a Pearson’s correlation coefficient of 0.6 (with the outlier included; open triangle) or 0.88 (with the outlier excluded). The mean concentration ranges in mouse versus human skin were similar (Figure 1D) indicating that mouse is a suitable model for drug testing increasing the relevance and translatability of mouse data.

## Discussion

To our knowledge this is the first study to demonstrate that tacrolimus can be measured in the skin of persons taking oral tacrolimus. In this small study it was demonstrated that the skin concentration of tacrolimus correlated with the blood concentration. Notably, several patients had multiple skin excisions taken from different sites and largely all the samples from each individual demonstrated a comparable skin tacrolimus concentration.

In this study, the tacrolimus blood level was collected in the early afternoon which is not the usual time that a trough level would be collected. This blood measurement was specifically collected to compare to the measured skin concentration of tacrolimus and determine whether there was any correlation. Higher tacrolimus concentrations were generally seen in the samples taken from patients’ faces (i.e. patients 8, 10, 12, 14, 15). However, it is not clear from this small data set whether or not areas more prone to Sun exposure exhibit higher skin concentrations of tacrolimus. We also assume there was a variable amount of fat in each sample depending on the anatomical location and this may also affect the pharmacokinetic distribution of tacrolimus.

Previous studies have measured tacrolimus concentration in the skin after topical application of tacrolimus [16]. A study comparing the delivery systems for topical tacrolimus measured tacrolimus concentration in human skin by liquid chromatography tandem-mass spectrometry. They also detected inflammatory markers such as IL-6 and IL-8. The researchers found that extensive barrier disruption resulted in the enhanced penetration of topically applied tacrolimus and uptake by immune cells in the skin. Other studies have measured the blood concentration of tacrolimus post skin application [17]. One study, using 0.1% tacrolimus ointment, found that systemic exposure tended to increase proportionally as the size of the treated body surface area increased, however the highest blood level was only 3% of the usual tacrolimus level measured in the blood of liver transplant patients receiving tacrolimus orally.

Given the significant issue of skin cancer post-transplant, effective therapies are urgently required to address this problem. Encouragingly new therapies and techniques are being actively studied to reduce the incidence of post-transplant skin cancer. A recent randomized, double-blind, placebo-controlled, single-arm trial explored whether topical sirolimus would reduce the incidence of skin cancer in solid organ transplant recipients with a history of skin cancer [18]. Participants had topical sirolimus applied to one forearm and hand for 12 weeks. At 12 weeks, the number of keratotic lesions had reduced in each patient by 31 +/- 5% and at 24 months there was a 3-fold decrease in intraepithelial carcinomas, however no difference in squamous cell carcinoma numbers were observed.

Our previous studies in mice show that a simple solution-based Q-2361 topical formulation achieved high (>30 μg/g) and sustained residence in skin with negligible drug levels in the blood [13]. In the current study, it was determined that the range of tacrolimus skin concentrations varied from 7.1 ng/g to 71.2 ng/g in patients receiving a total daily tacrolimus dose of between 1- and 12 mg of tacrolimus daily. It is entirely feasible, therefore, that appropriately formulated and topically applied Q-2361 could result in levels of Q-2361 over tacrolimus needed to locally rescue T cell function in patient skin. Furthermore, as a result of the correlation between blood and skin tacrolimus levels, the level of tacrolimus in patient skin can be approximated from routine blood analysis, without subjecting patients to additional skin biopsies, assisting with accurate dosing estimates for Q-2361 in future clinical trials.

Our study had several limitations. As the skin excisions were from different anatomical locations (depending on where the concern for malignancy was) the location and thickness of tissue was not standardized. It can also be assumed that different parts of skin have different degrees of Sun damage compared to others and we are uncertain of how that could affect tacrolimus concentrations. Finally, there was no power calculation attached to the chosen number of participants. Rather, recruitment ceased once there were enough numbers to see visual evidence of correlation between skin and blood levels.

Potential future research directions include sampling a larger cohort of patients and including the collection of information on race, skin type, and Sun exposure history. Collectively, this would allow for a deeper understanding of the variability in skin tacrolimus concentrations between different anatomical locations and among patients, and permit comparative analysis of skin tone, tacrolimus concentration, Sun exposure, and skin cancer prevalence.

There is an urgent unmet clinical need for new therapies to help address the issue of cutaneous malignancy in transplant recipients. This study importantly demonstrates that in mice and patients taking oral tacrolimus the drug concentrations can be measured in the skin and these levels appear to correlate between species and with blood tacrolimus concentrations in humans. Thus, this provides a rationale for the development of topical therapies such as Q-2361 which antagonize tacrolimus locally in the skin, aiming to reduce the development of skin cancer and potentially even treat established malignancy. Q-2361 is efficacious in mouse models of squamous cell cancer [13]. Clinical trials are now required to explore the efficacy of Q-2361 and whether individualization of the quantity and frequency of the treatment is required given the variability of tacrolimus skin concentrations between patients.

## Data Availability

The original contributions presented in the study are included in the article/supplementary material, further inquiries can be directed to the corresponding author.

## Abbreviations

DNA, deoxyribonucleic acid; FKBP12, 12-kDa FK506-binding protein; mTOR, mammalian target of rapamycin.
